# Limbal Pseudoepitheliomatous Hyperplasia Mimicking Ocular Surface Squamous Neoplasia in Palpebral Vernal Keratoconjunctivitis

**DOI:** 10.1155/2013/527230

**Published:** 2013-06-06

**Authors:** Chintan Malhotra, Arun K. Jain, Bikram Thapa

**Affiliations:** Advanced Eye Centre, Post Graduate Institute of Medical Education and Research (PGIMER), Sector 12, Chandigarh 160022, India

## Abstract

*Purpose*. Pseudoepitheliomatous hyperplasia at the limbus can mimic an ocular surface squamous neoplasia. It is an uncommon manifestation of vernal keratoconjunctivitis and has been reported previously in limbal VKC. It, however, has not been reported as a manifestation in the palpebral form of the disease and needs to be kept in the differential diagnosis of a limbal mass lesion in vernal keratoconjunctivitis. *Case Report*. We report the case of a 24 year old male patient having palpebral VKC and presenting with a papillomatous limbal mass with focal areas of keratinization mimicking an ocular surface squamous neoplasia. An excision biopsy was performed, and the specimen sent for histopathologywhich revealed features of pseudoepitheliomatous hyperplasia with no evidence of dysplasia or malignant transformation. The subepithelium revealed a dense plasma-rich inflammation. *Discussion*. We report this relatively uncommon presentation of limbal pseudoepitheliomatous hyperplasia mimicking an ocular surface squamous neoplasia in palpebral vernal keratoconjunctivitis. Wide excision as is required for an ocular surface neoplasia may thus be avoided if this entity is recognized in vernal keratoconjunctivitis.

## 1. Introduction

Vernal keratoconjunctivitis (VKC) is a chronic, bilateral conjunctival inflammatory condition with two distinct subtypes, palpebral and limbal. The palpebral form is associated with a hypertrophic tarsal response in the form of papillae, giving it the characteristic “cobblestone appearance” while the limbal form consists of diffuse thickening of the limbal tissue (usually at the superior limbus) and Horner Trantas spots. Localized involvement of the limbus in the form of a limbal mass is relatively uncommon. A hypertrophic limbal mass lesion in a case of limbal VKC has been reported previously [1]. We report a case of pseudoepitheliomatous hyperplasia (PEH) presenting as limbal mass and mimicking an ocular surface squamous neoplasia (OSSN) in a case of palpebral VKC.

## 2. Case Report

**Figure 1:**
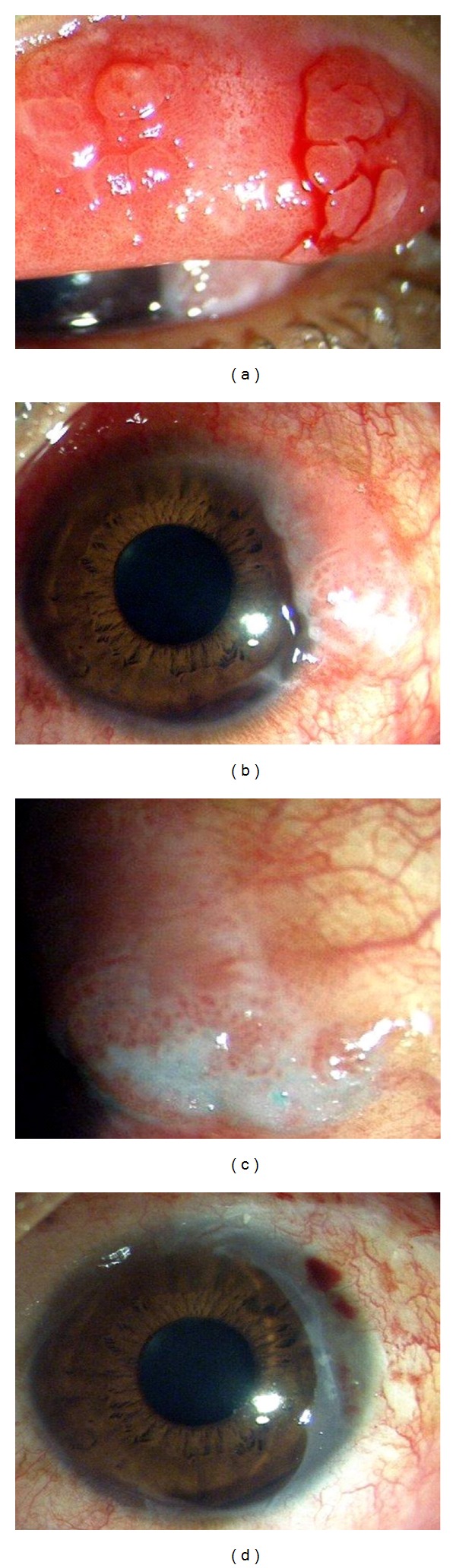
(a) Large cobblestone papillae on upper lid, left eye. (b) Limbal mass with vascular fronds on its surface. (c) A close-up view of the limbal mass—note the areas of keratinisation present on inferior border of the mass. (d) Amniotic membrane covering the conjunctival defect after excision biopsy.

**Figure 2:**
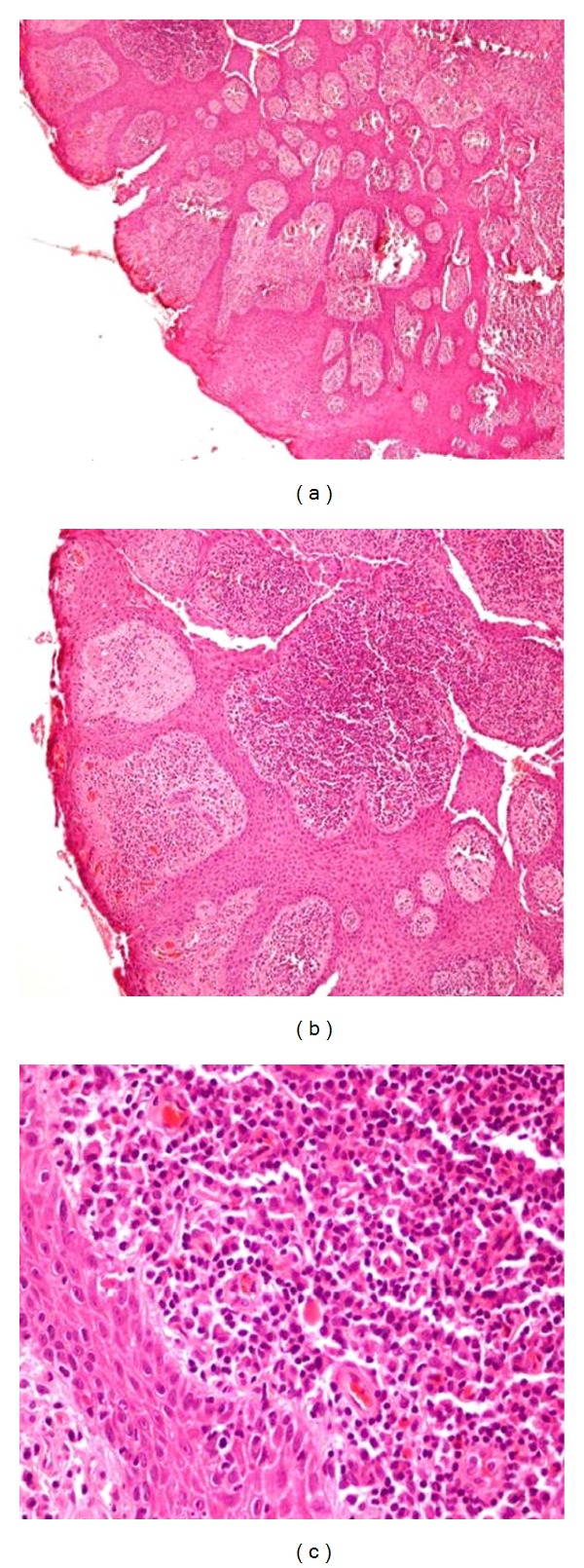
Haematoxylin and eosin stain. (a) The surface is lined by stratified squamous epithelium showing pseudoepitheliomatous hyperplasia. (×100). (b) The lining epithelium does not show any dysplasia or malignancy (×200). (c) The subepithelium shows dense plasma-cell-rich inflammation (×400).

A 24 year old male was examined in the cornea services of our institute in January 2013 with the chief complaint of noticing a mass in the left eye for the last 6 months which was increasing slowly in size. He also complained of severe itching in both eyes for many years for which he had been prescribed various topical medications. He underwent a complete ophthalmic examination bilaterally. Ocular examination revealed an unaided visual acuity of 20/20 OU and intraocular pressures of 16 and 18 mm of Hg in OD and OS, respectively, by Goldman applanation tonometry. Anterior and posterior segment ocular examination OD was unremarkable except for the presence of papillae on the upper palpebral conjunctiva. There was no limbal involvement in the right eye. On eversion of the left upper lid, an intense papillary reaction was seen diffusely associated with several large “cobblestone” papillae measuring 1.5–2 mm in the centre and lateral side of the lid (Figure 1(a)). These were associated with a ropy discharge. The temporal limbus in the left eye revealed the presence of a reddish sessile papillary mass extending for 2 hours from 2 to 4 o'clock (Figure 1(b)). The surface showed the presence of multiple capillary fronds appearing as red dots, with focal areas of keratinization on the inferior border of the lesion (Figure 1(c)). The mass was freely movable and the temporal edge of the mass was associated with engorged conjunctival vessels. The rest of the limbus did not show any evidence of thickening, and anterior and posterior segment examination was noncontributory. The surface of the mass stained significantly with rose bengal. Clinically, the diagnosis of bilateral, though asymmetric, palpebral VKC (left eye being more involved than the right) was made. For the limbal mass in the left eye, the possibility of epithelial hyperplasia was entertained but OSSN could not be ruled out. Excision biopsy with wide margins was performed, and the defect was covered with amniotic membrane using fibrin glue. Histopathological examination of the excisional biopsy specimen revealed that the surface was lined by stratified squamous epithelium showing pseudoepitheliomatous hyperplasia (Figure 2(a)). The lining epithelium did not show any evidence of dysplasia or malignancy while the subepithelium revealed a dense plasma-rich inflammation (Figures 2(b) and 2(c)).

## 3. Discussion

Involvement of the limbus in VKC usually occurs at the superior limbus in the form of conjunctival thickening which often may have a gelatinous appearance. Schwab et al. [1] have previously reported the occurrence of a nodular limbal mass lesion in a case of limbal VKC which was associated with hypertrophic conjunctival thickening present superiorly. Histopathologically, this lesion consisted of hyperplastic epithelium and collagenous connective tissue in the subepithelial stroma with a large number of eosinophils, fibroblasts, lymphocytes, and plasma cells—features consistent with a hypertrophic response in VKC.

In the present case, VKC was predominantly of the palpebral type, with no other limbal involvement except for the localized limbal mass present temporally in the left eye. Clinically, the differential diagnoses of PEH and OSSN were entertained. PEH is a benign proliferation of the conjunctival or corneal epithelium which often occurs in response to some preexisting inflammation. It usually presents as a white elevated mass with a hyperkeratotic surface [2]. When arising at the limbus, it may be difficult to clinically differentiate it from OSSN. One differentiating feature is the lack of capillary fronds in PEH while these may be seen in cases of squamous neoplasia or papillomas [2]. PEH mimicking OSSN following cultivated limbal epithelial transplantation has previously been reported [3].

In our case, the associated intense inflammation because of VKC and the location of the limbal mass at the area in contact with the cobblestone papillae on the upper lid (possibly causing mechanical irritation at that site) favoured the possibility of PEH. However, the presence of vascular fronds on the surface of the mass, the associated dilated conjunctival vessels on the lateral edge, and the staining with rose bengal raised the suspicion of OSSN. Histopathology was consistent with a diagnosis of PEH with no features suggestive of malignancy. We hypothesize that the localized epithelial hyperplasia could have occurred in our case because of the combination of chronic underlying inflammation aggravated by mechanical microtrauma to the limbal epithelium caused by rubbing of the large cobblestone papillae present on the upper lid. However, the opposite may also be true; that is, a limbal lesion may have been induced initially by irritation from superior existing papillae, followed by secondary mechanical giant papillary conjunctivitis (GPC) of the upper lid as the limbal lesion grew. This could be supported by the unusual focal nature of the larger papillae on the upper lid. GPC has been previously reported to occur because of mechanical and irritative etiologies [4]. Possibly a combination of the two mechanisms led to the final clinical picture in our case.

This case represents a secondary limbal involvement in a case of palpebral VKC. Awareness of this association of PEH presenting as a limbal mass in predominantly palpebral VKC and clinically mimicking an OSSN is important for ophthalmologists as PEH is a benign condition and does not require excision with large margins as is the case for OSSN [3].
